# Frequent expression loss of Inter-alpha-trypsin inhibitor heavy chain (ITIH) genes in multiple human solid tumors: A systematic expression analysis

**DOI:** 10.1186/1471-2407-8-25

**Published:** 2008-01-28

**Authors:** Alexander Hamm, Juergen Veeck, Nuran Bektas, Peter J Wild, Arndt Hartmann, Uwe Heindrichs, Glen Kristiansen, Tamra Werbowetski-Ogilvie, Rolando Del Maestro, Ruth Knuechel, Edgar Dahl

**Affiliations:** 1 Institute of Pathology, University Hospital of the RWTH Aachen, Aachen, Germany; 2 Institute of Pathology, University Hospital Zürich, Zürich, Switzerland; 3 Institute of Pathology, University of Erlangen, Erlangen, Germany; 4 Department of Gynecology, Breast Surgery and Mastology, University Hospital of the RWTH Aachen, Aachen; 5 McMaster Stem Cell and Cancer Research Institute (SCCRI), McMaster University, Faculty of Health Sciences, Hamilton, Ontario, Canada; 6 Brain Tumour Research Centre, Montreal Neurological Institute and Hospital, McGill University, Montreal, Quebec, Canada

## Abstract

**Background:**

The inter-alpha-trypsin inhibitors (ITI) are a family of plasma protease inhibitors, assembled from a light chain – bikunin, encoded by *AMBP *– and five homologous heavy chains (encoded by *ITIH1*, *ITIH2*, *ITIH3*, *ITIH4*, and *ITIH5*), contributing to extracellular matrix stability by covalent linkage to hyaluronan. So far, ITIH molecules have been shown to play a particularly important role in inflammation and carcinogenesis.

**Methods:**

We systematically investigated differential gene expression of the *ITIH *gene family, as well as *AMBP *and the interacting partner *TNFAIP6 *in 13 different human tumor entities (of breast, endometrium, ovary, cervix, stomach, small intestine, colon, rectum, lung, thyroid, prostate, kidney, and pancreas) using cDNA dot blot analysis (Cancer Profiling Array, CPA), semiquantitative RT-PCR and immunohistochemistry.

**Results:**

We found that *ITIH *genes are clearly downregulated in multiple human solid tumors, including breast, colon and lung cancer. Thus, *ITIH *genes may represent a family of putative tumor suppressor genes that should be analyzed in greater detail in the future. For an initial detailed analysis we chose *ITIH2 *expression in human breast cancer. Loss of *ITIH2 *expression in 70% of cases (n = 50, CPA) could be confirmed by real-time PCR in an additional set of breast cancers (n = 36). Next we studied ITIH2 expression on the protein level by analyzing a comprehensive tissue micro array including 185 invasive breast cancer specimens. We found a strong correlation (p < 0.001) between ITIH2 expression and estrogen receptor (ER) expression indicating that ER may be involved in the regulation of this ECM molecule.

**Conclusion:**

Altogether, this is the first systematic analysis on the differential expression of *ITIH *genes in human cancer, showing frequent downregulation that may be associated with initiation and/or progression of these malignancies.

## Background

The inter-alpha (globulin) inhibitor (ITI) family (more commonly called the family of inter-alpha-trypsin inhibitors) is composed of serine protease inhibitors that are assembled from two precursor proteins: a light chain and either one or two heavy chains[1,2]. While there is only one type of light chain, there are different homologous heavy chains (ITIHs), to date consisting of five members (Table 1).

**Table 1:** Family of human Inter-alpha-Inhibitor genes (and TNFAIP6)

**Official Symbol**	**Official Name**	**Other Aliases**	**Chromosomal Localisation**
*ITIH1*	inter-alpha (globulin) inhibitor H1	H1P, IATIH, IGHEP1, Inter-alpha-inhibitor heavy chain 1, Inter-alpha-trypsin inhibitor complex component III, Inter-alpha-trypsin inhibitor heavy chain H1 precursor, ITIH, ITI heavy chain H1, Serum-derived hyaluronan-associated protein, SHAP	3p21.2-p21.1
*ITIH2*	inter-alpha (globulin) inhibitor H2	H2P, IGHEP2, Inter-alpha-inhibitor heavy chain 2, Inter-alpha-trypsin inhibitor complex component II, Inter-alpha-trypsin inhibitor heavy chain H2 precursor, ITI heavy chain H2, Serum-derived hyaluronan-associated protein, SHAP	10p15
*ITIH3*	inter-alpha (globulin) inhibitor H3	Inter-alpha-inhibitor heavy chain 3, Inter-alpha-trypsin inhibitor heavy chain H3 precursor, ITI heavy chain H3, Serum-derived hyaluronan-associated protein, SHAP	3p21.2-p21.1
*ITIH4*	inter-alpha (globulin) inhibitor H4 (plasma Kallikrein-sensitive glycoprotein)	GP120, H4P, IHRP, Inter-alpha-inhibitor heavy chain 4, Inter-alpha-trypsin inhibitor family heavy chain-related protein, Inter-alpha-trypsin inhibitor heavy chain H4 precursor, ITI heavy chain H4, ITIHL1, PK120, PK-120, Plasma kallikrein sensitive glycoprotein 120	3p21-p14
*ITIH5*	inter-alpha (globulin) inhibitor H5	Inter-alpha trypsin inhibitor	10p15
*AMBP*	alpha-1-microglobulin/bikunin precursor	AMBP protein precursor, HCP, ITI, ITIL, UTI	9q32-q33
*TNFAIP6*	tumor necrosis factor, alpha-induced protein 6	Hyaluronate-binding protein, TNF-stimulated gene 6 protein, TSG6, Tumor necrosis factor-inducible protein TSG-6 precursor	2q23.3

Official and alias names as found on the National Library of Medicine Website [48].

The light chain is encoded by alpha-1-microglobulin/bikunin precursor (*AMBP*), which also codes for alpha-1-microglobulin, a member of the lipocalin superfamily that is not functionally or structurally related to the ITI family[3]. ITI light chain contains two tandem-repeats of kunitz type domains and has thus been assigned the name "bikunin"[4]. The family of heavy chains (ITIHs), on the opposite, is encoded by five genes located on two different chromosomes [5-7]: *ITIH1*, *ITIH2*, *ITIH3*, *ITIH4*, and *ITIH5*. Of these, *ITIH1*, *ITIH3*, and *ITIH4 *map to a closely linked region on chromosome 3p21 [6] whereas *ITIH2 *und *ITIH5 *are tandemly arranged on chromosome 10p15[7]. During assembly of the mature ITI protein in the liver, the precursor proteins for ITIH1-3 and bikunin undergo extensive posttranslational modifications[8], mainly involving trimming of the C-terminal ends [3]. However, the conserved cleavage site of these heavy chains is absent in ITIH4[3], thus preventing a bond with bikunin. Interestingly, the heavy chains (mostly ITIH1 and ITIH2) are linked to bikunin via a single chondroitin sulfate chain[1,9], making ITI a both structurally and functionally unique proteoglycan with a plasma protease inhibitory activity [9], which resides solely in the bikunin part of the molecule[3]. On the other hand, the only function known so far of the heavy chains is the covalent linkage to hyaluronic acid (HA)[10], which is a major component of the extracellular matrix (ECM), but is also secreted into body fluids, such as blood and lymph fluid. The transfer of the ITI heavy chains – due to this linkage also called serum-derived hyaluronan-associated protein (SHAP)[10] – onto HA requires tumor necrosis factor alpha induced protein 6 (TNFAIP6), also known as TNF stimulated gene (TSG-6) [11]. TNFAIP6 not only potentiates the anti-plasmin activity of ITI [12], but forms a stable complex [13] with ITIH and HA during the transesterification reaction[14], specifically[15] enhancing the transfer of the heavy chains as a catalytic factor in the presence of calcium ions [16] (for a review on TNFAIP6 see Ref. [17]). In addition, the bikunin-chain is required for the linking process in the sense of a "SHAP-presenting molecule"[2]. The main function of ITIH is proposed to be an essential factor in the stabilization of the ECM[1] (extensively investigated in cumulus oophorus cells[18]), based on the covalent linkage of HA to the heavy chain, producing so-called "cable-like structures"[2].

For a long period of time, the focus of attention has been the urinary trypsin inhibitory activity, which originally led to the discovery of the ITI molecule. An influence on calcium oxalate cristallization and other renal and postrenal processes has been investigated in depth[19]. In the past two decades, however, the role of ITI in a broad range of both physiological and pathological conditions could be elucidated. Although its specific plasma-proteinase inhibitory activity accounts for only 5% of the total proteinase inhibitors[20], a strong involvement of ITI in inflammation as well as in carcinogenesis and metastatic processes can be postulated based on the published data available. In the inflammation setting, ITI family members have been demonstrated to be both positive and negative acute phase proteins[2] under various conditions such as pancreatitis[21], polyarthritis[22], myocardial infarctions[23], colitis[24], or sepsis[25,26], presenting possibilities for diagnostic and therapeutic purposes (e.g. administration of ITI in systemic sepsis[25,27] or as a protective agent in anthrax intoxication[28]). On the other hand, there is strong evidence that all members of the ITI family play an important role in different aspects of malignant processes. The proteinase inhibitory activity of bikunin suggests an influence on cell growth and metastasis of tumor cells[1,29-31], as especially cell-bound plasmin activity has been shown to play a key role in both degradation of the ECM[32] and angiogenesis[33]. The ITI heavy chains, as described above, most effectively stabilize the ECM and have been shown to be involved in processes such as tumor invasion[34] and metastasis [35]. For example, ITIH1 and ITIH3 have been shown to increase cell attachment *in vitro *and to reduce the number of metastases in a murine *in vivo *model [36]. In addition, ITIH5 has been proven to be a novel prognostic marker in invasive node-negative breast cancer, demonstrating its involvement in tumor progression, invasion, and metastasis[37].

Although these results clearly suggest that all members of the ITI family may contribute to carcinogenesis via deregulated gene expression (hence influencing most important cellular regulation mechanisms, such as proliferation, differentiation, apoptosis, and extracellular matrix stability), to date there has been no approach to systematically investigate differential gene expression of the ITI family in human cancer. In the present study, we address the issue of up- or downregulation of ITI family genes, focussing on the five heavy chains, their light chain partner bikunin (encoded by *AMBP*) and their important functional regulator TNFAIP6. Using cancer profiling arrays (CPAs), we quantitatively analyzed expression of these seven genes in a large panel of normal and malignant human tissues. We show that the expression patterns of ITIH molecules are clearly deregulated in a variety of human cancers, providing further evidence for their potential role as tumor suppressor and/or metastasis repressor genes.

## Methods

### Clinical Materials

Samples of breast cancer specimens (n = 36) for real-time PCR analysis were obtained from patients treated by primary surgery for breast cancer at the University Hospitals of Aachen, Jena and Regensburg, Germany. All patients gave informed consent to participate in the study. Tumor material was snap-frozen in liquid nitrogen immediately after surgery. Hematoxylin and eosin stained sections were prepared for assessment of the percentage of tumor cells, only samples with greater than 70% tumor cells were selected for analysis. Frozen tissue samples were homogenized in liquid nitrogen and dissolved in lysis buffer followed by RNA isolation using TRIzol (Gibco-BRL, Glasgow, UK) according to the protocol supplied by the manufacturer. Clinicopathological data are presented in Table 2.

**Table 2:** Clinicopathological and immunohistochemical characteristics of primary breast carcinomas

**Variable**	**Categorization**	**IHC**		**Realtime PCR**	
		**n^1^**	**%**	**n^1^**	**%**

***Clinicopathological factor:***					
Age at diagnosis					
Median, range (years)		57.5 (25–82)		57.5 (28–85)	
	≤ 50 years	54	*29.2*	10	*27.8*
	> 50 years	128	*69.2*	26	*72.2*
	unknown	3	*1.6*	0	*0*
Tumor stage^2^					
	pT1	46	*24.9*	15	*41.7*
	pT2	92	*49.7*	16	*44.4*
	pT3	13	*7.0*	0	*0*
	pT4	30	*16.2*	4	*11.1*
	pTx^3^	4	*2.2*	1	*2.8*
Lymph node status^2^					
	pN0	71	*38.4*	19	*52.8*
	pN1–3	104	*56.2*	15	*41.7*
	pNx^3^	10	*5.4*	2	*5.6*
Grading					
	G1/G2	100	*54.1*	19	*52.8*
	G3	80	*43.2*	15	*41.7*
	Gx^3^	5	*2.7*	2	*5.6*
Histological type					
	ductal	145	*78.4*	34	*94.4*
	lobular	13	*7.0*	1	*2.8*
	other	19	*10.3*	1	*2.8*
	unknown	8	*4.3*	0	*0*
					
***Immunohistochemistry (IHC):***					
Estrogen receptor status					
	negative (IRS^4 ^0–2)	49	*26.5*	11	*30.6*
	positive (IRS 3–12)	97	*52.4*	21	*58.3*
	unknown	39	*21.1*	4	*11.1*
Progesterone receptor status					
	negative (IRS^4 ^0–2)	107	*57.8*	12	*33.3*
	positive (IRS 3–12)	49	*26.5*	20	*55.6*
	unknown	29	*15.7*	4	*11.1*
HER2 expression status					
	negative (0–1+)	120	*64.9*	6	*16.7*
	positive (2+-3+)	38	*20.5*	5	*13.9*
	unknown	27	*14.6*	25	*69.4*

Characteristics of primary breast carcinomas used for ITIH2 immunohistochemistry (n = 185) and real-time PCR (n = 36) analysis.

^1^Only female patients with primary invasive breast cancer were included. ^2^According to UICC: TNM Classification of Malignant Tumours (2002) [65]. ^3^x = status unknown. ^4^IRS = Immunoreactivity score according to Remmele and Stegner (1987) [47].

A breast cancer tissue microarray was constructed as described before [38,39], including 28 normal tissue samples, 185 invasive carcinomas, and 2 carcinomas *in-situ*. All patients gave informed consent to participate in the study. Clinical follow-up, provided by the Central Tumor Registry Regensburg, Germany, was available for all breast cancer patients with a median follow-up period of 79 months (0–148 months). Clinicopathological data are presented in Table 2.

### RNA expression analysis using Multiple Tissue Northern Blot – MTN

Tissue specific expression of each gene was analyzed using Human Multiple Tissue Northern Blot I and Blot II (Product no. 636806 and 636805, respectively; Clontech, Heidelberg, Germany). Each MTN consisted of eight lanes from different human tissues, containing approximately 2 μg of poly A+ RNA per lane. RNA was run on denaturing formaldehyde 1.0% agarose gels, transferred to a nylon membrane by Northern blotting, and fixed by UV irradiation. Lanes 1–8 on MTN I contain, in order, RNA from 1) heart, 2) brain, 3) placenta, 4) lung, 5) liver, 6) skeletal muscle, 7) kidney, 8) pancreas. Lanes 1–8 on MTN II contain, in order, RNA from 1) spleen, 2) thymus, 3) prostate, 4) testis, 5) ovary, 6) small intestine, 7) colon (no mucosa), 8) peripheral blood leukocytes (PBL).

cDNA probes for hybridization were generated by PCR using commercially available normalized cDNA panels derived from different human tissues (Product no. 636742, Clontech, Heidelberg, Germany[40]). RT-PCR was performed on a Peltier Thermal Cycler-200 (Biozym, Hamburg, Germany), using intron-spanning primers. Primers and cDNAs used for each probe are presented in Table 3. Amplified cDNA fragments were purified using the QIAquick PCR purification Kit (Qiagen, Hilden, Germany) in order to obtain the gene-specific cDNA probes.

**Table 3:** Primers used to generate cDNA probes for dot blot hybridization

**Gene**	**Primers**	**Product Size (bp)**	**Tissue Source**
*ITIH1*	5'-AAA GGG TCA TGT GCT GTT CC-3'	1121	liver
	5'-ACC CAT AGT CCA GCG ACA TC-3'		
*ITIH2*	5'-TGT TCA GAT CCC CAA AGG AG-3'	1169	liver
	5'-ATG GAG TGG AGA CCT GGT TG-3'		
*ITIH3*	5'-GCT GAG GCC TCT TTC ATC AC-3'	1019	liver
	5'-TCC TTC ATG TCC ACC TCC TC-3'		
*ITIH4*	5'-CTT CAA GGG CTC AGA GAT GG-3'	1141	liver
	5'-GTC AGT GTC ACG CAG AAG GA-3'		
*ITIH5*	5'-GAG GCC AAG TCT GCA TCT TC-3'	1010	placenta
	5'-GAT GAC TCT GCT CGG TGT GA-3'		
*AMBP*	5'-AGC TCC TCA TCA CCA TCA CC-3'	913	liver
	5'-TTC TTC ACC AGC TGC TCC TT-3'		
*TNFAIP6*	5'-AAG GAT GGG GAT TCA AGG AT-3'	781	skeletal muscle
	5'-TGG CTA AAT CTT CCA GCT AAA AA-3'		

Hybridization was then performed using 25 ng of the gene-specific ^32^P-labeled cDNA probes. These gene-specific cDNA fragments were radiolabeled using a Megaprime labeling kit (Amersham Biosciences, Braunschweig, Germany), hybridized overnight at 65°C using ExpressHyb Hybridization Solution (Clontech, Heidelberg, Germany), washed, and exposed to Kodak XAR-5 X-ray film with an intensifying screen (Eastman Kodak Co., Rochester, NY, USA).

The specificity of each hybridization probe was determined by the co-hybridization of nylon membranes containing different concentrations of spotted cDNA probe: 20 pg, 2 pg, and 0.2 pg of cDNA from each gene were diluted in 3 μl of 20 × SSC buffer, heat-denatured for 5 min by boiling and then quenched on ice. Denatured cDNAs were spotted on Hybond N+ membranes (Amersham Biosciences, Freiburg, Germany) and fixed by UV irradiation for 3 minutes. These membranes were treated during filter hybridization, washing and exposition exactly like the MTNs.

### Expression analysis using Cancer Profiling Array – CPA

Expression of the five heavy chain genes (*ITIH1*, *ITIH2*, *ITIH3*, *ITIH4*, and *ITIH5*) and the light chain gene (*AMBP*), as well as *TNFAIP6 *were analyzed using the Cancer Profiling Array (Product No. 631761; Clontech, Heidelberg, Germany) [41-43], containing spotted tumor cDNAs and corresponding normal tissue from the same patient [44].

The Cancer Profiling Array (CPA) consisted of 511 dots with 494 cDNAs synthesized from various human tumors and corresponding normal tissue specimens, i.e. 241 tumor and 241 matched normal tissue specimens as well as 12 cDNAs from metastases corresponding to 12 of the tumor/normal pairs. The following 241 matched tumor/normal tissue cDNA pairs and 12 matching metastases were included on the CPA: 50 breast cancer/50 normal/three matching metastases, 42 uterine cancer/42 normal/two matching metastases, 35 colon cancer/35 normal/four matching metastases, 27 gastric cancer/27 normal stomach, 14 ovarian cancer/14 normal/two matching metastases, one cervical cancer/one normal, 21 lung cancer/21 normal, 20 renal cancer/20 normal, 18 rectal cancer/18 normal/one matching metastase, two small intestine cancer/two normal, six thyroid cancer/six normal, four prostate cancer/four normal, one pancreatic cancer/one normal. Each cDNA pair was independently normalized based on the expression of four housekeeping genes (ubiquitin, 23 kDa highly basic protein, β-actin and glutamate dehydrogenase) and immobilized in separate dots. Patient age, histological type, disease stage, tumor size, node status, and presence or absence of metastases for each specimen is supplied with the product and can be obtained from the manufacturer upon request.

Hybridization of CPA was performed as described above for the Multiple Tissue Northern blot. The tumor/normal intensity ratio was calculated using a Typhoon 9410 High Performance Imager (GE-Healthcare, Chalfont St. Giles, UK) and normalized against the background. We defined a gene as differentially expressed in a given tumor entity if a common deregulation (two-fold up- or down-regulation according to the well-established fold change two approach, FC2) was detectable in at least 50% of tumor tissue samples analyzed. Fold changes between 0.5 and 2.0 were considered as not differentially expressed.

### Semiquantitative Real-Time PCR

Semiquantitative PCR was performed using the LightCycler system together with the LightCycler DNA Master SYBR Green I Kit (Roche Diagnostics, Basel, Switzerland). Reaction volumes of 20 μl consisted of the following components: 25 mM MgCl_2_, 10 μM forward primer, 10 μM reverse primer, 2 μl LightCycler DNA Master SYBR Green I and 1 μl of cDNA as PCR template. Gene expression was quantified by the comparative C_T _method, normalizing C_T_-values to the housekeeping gene *GAPDH *and calculating relative expression values[45].

Primer sequences for *ITIH2 *expression analysis were: forward 5'-ACC AGG TCT CCA CTC CAT TG-3'; reverse 5'-ATC CTG CAA GTC GTC CAT CT-3' (230 bp product size) and for the reference gene *GAPDH*: 5'-GAA GGT GAA GGT CGG AGT CA-3'; reverse 5'-TGG ACT CCA CGA CGT ACT CA-3' (108 bp product size). The cycling conditions were set up to an initial denaturation at 95°C for 15 min, followed by 40 cycles with denaturation at 95°C for 20 s, annealing at 60°C for 20 s and elongation at 72°C for 30 s. To verify the specificity of the PCR products, melting curve analyses were performed. The relative gene expression levels were standardized to the expression level of a normal breast tissue sample that contained approximately 50% of epithelial cells (tumors generally contained >70% of tumor cells). To ensure experiment accuracy, all reactions were performed in triplicates.

### Immunohistochemistry

Whole tissue sections or tissue microarrays (TMAs), respectively were stained with ITIH2 antibody which has been published before[46]. Briefly, ITIH2 antibodies directed against the C-terminal region of ITIH2 protein were generated by immunizing rabbits with the synthetic oligopeptide PGKDPEKPEASMEVK coupled to KLH (Sheldon Biotechnology Centre, McGill University, Montreal, Canada). For immunohistochemistry, tissue sections were deparaffinized in xylene, rehydrated in a decreasing ethanol series and pre-incubated with peroxidase blocking solution. Staining with ITIH2 antibody was performed in a dilution of 1:600 (no pre-treatment for antigen retrieval), followed by a second-step incubation with Dako's HRP, Rabbit/Mouse (ENV) reagent, using the Dako REAL™ EnVision™ Detection System K5007 (Dako, Glestrop, Denmark). Reaction was visualized by Dako REAL™ DAB+ Chromogen. Sections were counterstained with hematoxylin, dehydrated in an increasing ethanol series and mounted with Vitro Clud (Langenbrinck, Emmendingen, Germany) The application of primary antibodies was omitted in negative controls, while liver sections (as tissue with the strongest ITIH2 expression) were used as positive controls. Sections were examined and scored by a pathologist, using a semiquantitative immunoreactive score (IRS) as described previously[47]. Mean dye intensity was assessed using the following scale: 0, negative; 1, low; 2, middle; and 3, strong. The percentages of stained cells varied as follows: 0, negative; 1, <10%; 2, 10% to 50%; 3, 51% to 80%; 4, >80% positive cells. The product of both summands yields a total score ranging from 0 to 12 points. According to the scores, tissues were classified as having weak (0–4 points) or strong (6–12 points) ITIH2 expression.

### Statistical analysis of clinicopathological patient data

Statistical analyses were completed using SPSS version 14.0 (SPSS, Chicago, IL, USA). Differences were considered statistically significant when P values were <0.05. Cross tables with clinicopathological characteristics *versus *ITIH2 staining were established, using Fisher's exact test (two-sided) to evaluate significancy. Overall survival (OS) and recurrence-free survival (RFS) curves comparing patients with strong *versus *weak staining in immunohistochemistry analysis were calculated using the Kaplan-Meier method, with significance evaluated by two-sided log-rank statistics. OS and RFS were measured from time of surgery until tumor-related death or recurrence, respectively. Patients were censored at the time of their last tumor-free clinical follow-up appointment or at their date of death not related to the tumor.

## Results

### Expression analysis of the ITIH gene family in 13 different human tumor entities and normal human tissues

**Figure 1:**
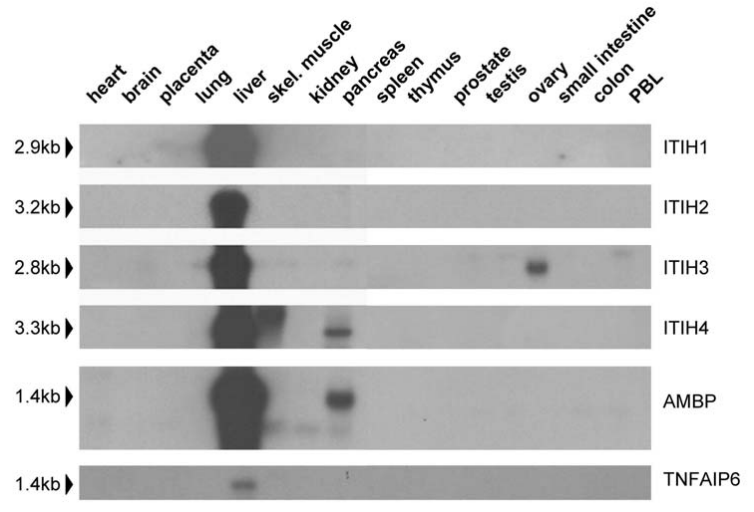
**Expression of the ITIH gene family and AMBP and TNFAIP6 genes in normal human tissues**. RNA expression analysis using Clontech's Multiple Tissue Northern (MTN) blot demonstrates specificity and correct transcript size of the respective gene probes later used for dot blot analysis. See text for details.

Expression of ITIH gene family members (*ITIH1*, *ITIH2*, *ITIH3*, *ITIH4*, and *ITIH5*) as well as two additional genes (*AMBP *and *TNFAIP6*) encoding proteins that regulate ITIH functions were analyzed in human benign and malignant tissues by two different methods. First, each of the gene probes (except for *ITIH5*, which we have published before[7]) was hybridized to a Northern blot containing poly A+ RNA derived from 16 different human normal tissues (Multiple Tissue Northern Blot – MTN) to determine its transcript size(s) and the specificity of the probes later used for cDNA dot blot hybridization. Figure 1 demonstrates that *ITIH *mRNAs are predominantly expressed in the liver with the exception of *ITIH5*, which is most abundantly expressed in placental tissue[7]. *ITIH3 *and *ITIH4*, in addition, show abundant expression in ovarian and pancreatic tissue, respectively. All six probes presented a specific hybridization signal on Northern blot and the determined sizes of the corresponding mRNA transcripts were in good accordance with mRNA sizes annotated on the National Library of Medicine Webpage [48].

**Figure 2:**
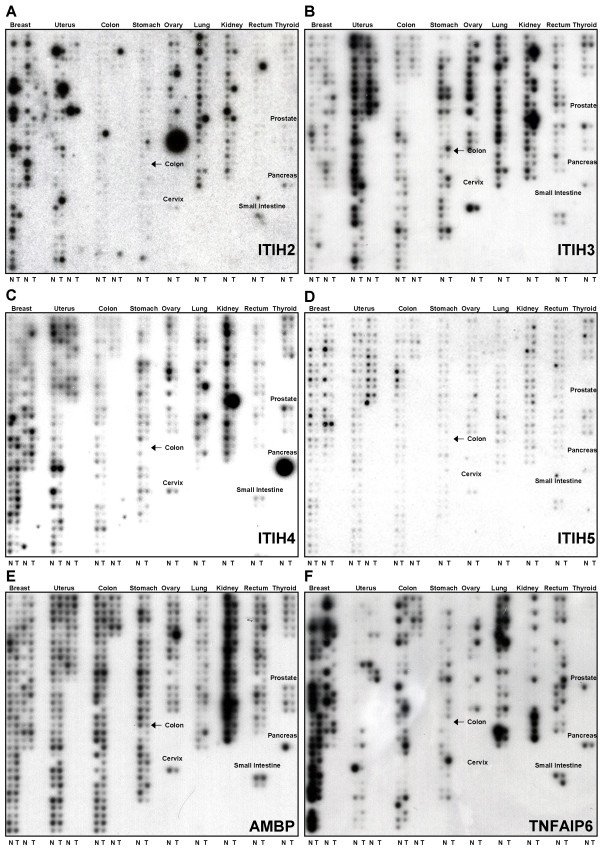
**Differential expression of the ITIH gene family in 13 different human tumor entities**. Gene probes for *ITIH *genes, as well as the *AMBP *and *TNFAIP6 *genes, were hybridized to a Cancer Profiling Array (Clontech, Heidelberg, Germany) containing spotted cDNAs from 241 human tumors and 241 corresponding human normal tissues. See text for details and Table 4 for a detailed densitometric evaluation of the hybridization signals.

Next, these gene probes were used for dot blot hybridization on a Cancer Profiling Array (CPA). The CPA contains spotted cDNAs from 241 tumor and 241 matched normal tissue samples representing 13 different human tumor entities. These dot blot arrays are highly sensitive in the detection of even rare transcripts since a considerable amount of cDNA (10–50 μg) has been spotted in a very small area (1 mm in diameter). In analysis of differential expression, the percentage of up-regulation or down-regulation was defined according to the well-established fold change two approach (FC2, see Material and Methods). Interestingly, each member of the *ITIH *gene family except *ITIH1 *(data not shown) exhibited highly differential expression in a remarkable number of human tumor entities (Figure 2). These differential expression patterns nearly exclusively presented as loss of *ITIH *mRNAs in tumor tissues. An overview of the complete expression data is provided in Table 4. In contrast to the highly differential expression of *ITIH *genes, *AMBP *and *TNFAIP6 *exhibited a much broader expression pattern with differential gene expression restricted to kidney tissue. In detail, the expression patterns of the analyzed genes on the CPA presented as follows:

**Table 4:** Frequent loss of ITIH expression in multiple human solid tumors.

**Tissue**	**n**	**ITIH1 regulation (%)**		**ITIH2 regulation (%)**		**ITIH3 regulation (%)**		**ITIH4 regulation (%)**		**ITIH5 regulation (%)**		**AMBP regulation (%)**		**TNFAIP6 regulation (%)**	
		
		**up**	**down**	**up**	**down**	**up**	**down**	**up**	**down**	**up**	**down**	**up**	**down**	**up**	**down**
Breast	50	2	6	2	**70**	9	**51**	6	16	6	**52**	4	10	6	35
Uterus	42	0	10	12	26	5	**62**	7	29	5	24	0	7	21	13
Colon	35	11	9	11	43	9	**67**	9	**54**	3	40	3	0	31	31
Stomach	27	7	19	30	**56**	12	38	4	**63**	15	19	0	37	48	12
Ovary	14	7	14	18	27	21	**71**	14	**57**	0	21	21	7	43	29
Lung	21	0	0	0	**71**	0	**86**	10	**52**	0	14	5	5	0	30
Kidney	20	0	35	0	**70**	10	35	0	**95**	15	0	0	**90**	**91**	0
Rectum	18	0	11	22	**61**	6	**72**	6	**50**	11	22	6	11	13	25
Thyroid	6	0	0	17	17	0	**50**	17	33	0	17	0	17	n.e.	
Prostate	4	**50**	25	0	**50**	0	**100**	0	**75**	0	0	0	25	n.e.	

Densitometric analysis of hybridization signals shown in Fig. 2. Percentages of deregulation (two-fold up- or down-regulation according to the well-established fold change two approach, FC2) in the given tumor entity (n.e. = not expressed). Fold changes between 0.5 and 2.0 were considered as not differentially expressed and are represented as the missing percentages to 100% in each tumor entity. Only tumor entities with four or more samples (n ≥ 4) on the CPA were considered for densitometric evaluation.

*ITIH2 *mRNA expression was detectable in normal breast, uterus, ovary, lung and kidney tissues with little expression in the other eight tissues analyzed. Loss or downregulation of *ITIH2 *expression was seen in 70% of breast cancers, 71% of lung cancers, and 70% of renal tumors (see Figure 2A). Additionally, careful densitometric evaluation of the hybridization signals showed downregulation in 56% of gastric cancers, 61% of rectal carcinomas, and 50% of prostate cancers.

*ITIH3 *mRNA expression was detectable in normal breast, uterus, colon, stomach, ovary, lung, kidney and rectum (Figure 2B). Downregulation of *ITIH3 *expression was seen in cancers of the breast (51%), uterus (62%), colon (67%), ovary (71%), lung (86%), rectum (72%), and prostate (4 out of 4 samples).

*ITIH4 *mRNA was detectable in normal breast, uterus, stomach, ovary and lung (see Figure 2C). Expression is especially abundant in the normal kidney, very much in accordance with the Northern blot data (Figure 1) and thus clearly demonstrating that dot blot analysis of spotted cDNAs (CPA analysis) is much more sensitive than Northern blot hybridization of poly A+ RNAs. *ITIH4 *mRNA was only weakly expressed in normal colon, rectum, and small intestine. Downregulation of *ITIH4 *in cancer tissue was detectable in tumors of the kidney (95%), stomach (63%) and ovary (57%) as well as in colon cancer (54%), lung cancer (52%), rectum cancer (50%), and prostate cancer (75%).

*ITIH5 *mRNA was detectable in normal breast, uterus, colon and kidney. MTN expression data has been published before, showing strong expression in placenta and lower expression in mammary gland and ovary [7]. However, a significant downregulation of *ITIH5 *expression (52%) was observed in breast cancer only (Figure 2D).

*AMBP *is abundantly expressed in all 13 tissues represented on the CPA. A prominent downregulation of 90% was detectable in kidney cancer (Figure 2E). *TNFAIP6 *is very abundantly expressed in several normal tissues like breast, lung, colon, uterus and rectum. A highly significant upregulation of *TNFAIP6 *mRNA expression was observed in kidney cancer in 91% of cases (Figure 2F). A clear *TNFAIP6 *upregulation below the defined cut-off of 50% was furthermore detectable in tumors of the ovary and stomach (43% and 48%, respectively).

### Differential expression analysis of ITIH2 downregulation in breast cancer using Reverse Transcriptase PCR (RT-PCR)

**Figure 3:**
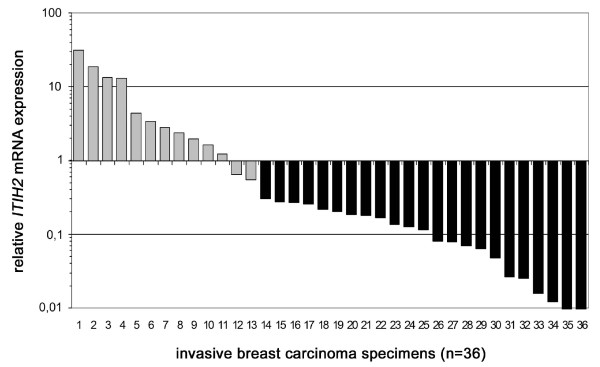
**ITIH2 mRNA expression analysis as determined by real-time PCR**. 36 breast carcinoma specimens were analyzed. Each tumor expression level was calculated as fold change expression *versus *a normal breast tissue cDNA (set to 1 on y-axis). 23/36 samples (64%) exhibited a substantial reduction of *ITIH2 *mRNA, indicated by an expression fold change of >2 in the tumor tissue, thus were scored as being downregulated (black bars) whereas grey bars represent specimens with non-deregulated or upregulated *ITIH2 *expression.

As the main focus of our workgroups lies on molecular understanding of breast cancer, we chose ITIH2 for a detailed expression analysis in human breast cancer. To provide further evidence that *ITIH2 *mRNA is differentially expressed in breast cancer we performed a semiquantitative real-time PCR analysis on a cohort of 36 primary breast carcinomas. The relative *ITIH2 *expression level of each specimen was normalized against expression of a non-malignant breast tissue cDNA and calculated as fold change-value of tumor tissue *versus *normal tissue. A fold change > 2 (FC2) was considered as being deregulation of normal *ITIH2 *expression. Due to this cut-off, 23 out of 36 breast tumor specimens exhibited *ITIH2 *downregulation (63.9%) by FC2, whereas 13 out of 36 specimens (36.1%) showed no deregulation or upregulation of *ITIH2 *(Figure 3). In line with the results derived from the CPA, *ITIH2 *mRNA downregulation in breast cancer could thus be confirmed by a second independent technique.

### Immunohistochemistry of ITIH2 expression in invasive carcinomas

**Figure 4:**
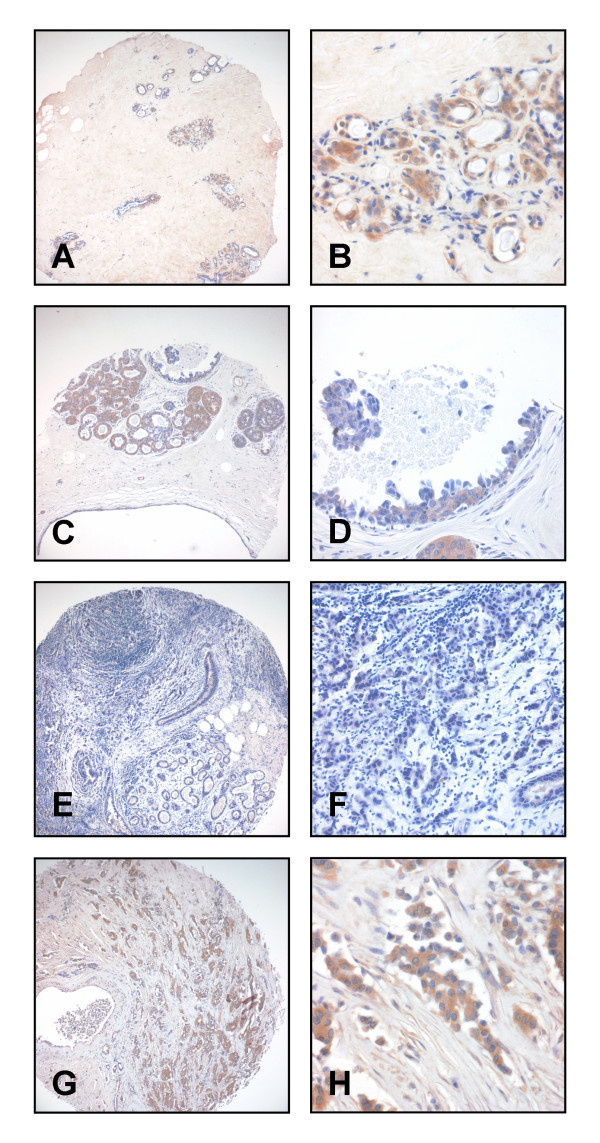
**ITIH2 immunohistochemistry on TMA derived from normal and cancerous breast tissue**. A+B: Strong cytoplasmic staining is seen in normal epithelial cells of the mammary gland. C+D: Ductal carcinoma *in-situ *(high grade type) with moderate focal cytoplasmic staining and normal, partially hyperplastic gland epithelium with strong cytoplasmic staining (see arrows). E-H: Invasive ductal carcinoma with either negative (E, F) or strong ITIH2 staining (G, H). Magnification: 100× (A, C, E, G), 400× (B, D, F, H).

Immunohistochemical analysis was applied to compare ITIH2 protein expression in normal and malignant breast tissue. A tissue microarray containing 185 invasive breast carcinomas, 2 carcinomas *in-situ *(DCIS), and 28 normal breast tissue samples was used. Intensity and quantity of immunohistochemical staining was evaluated using a semiquantitative immunoreactivity score (IRS) [47]. ITIH2 expression was clearly detectable in the epithelium of normal breast tissue (Figure 4A and 4B). ITIH2 expression was maintained in hyperplastic gland epithelium and ductal carcinoma *in-situ *(DCIS), however, ITIH2 expression was somewhat weaker in DCIS than in normal tissue (Figure 4C and 4D). In 44% (81/185) of invasive carcinomas of the breast, ITIH2 expression was strongly reduced or completely lost (Figure 4E and 4F) while 56% of invasive carcinomas (104/185) maintained moderate to strong ITIH2 expression (Figure 4G and 4H).

ITIH2 expression was also studied on whole tissue sections of invasive tumors. In case of abundant expression, ITIH2 was homogeneously distributed across the tumor masses and showed neither a patchy expression pattern, nor a particular concentration at the tumor invasion front (data not shown).

### Statistical analysis of ITIH2 expression

For descriptive data analysis, we associated clinicopathological data with ITIH2 expression status, categorizing ITIH2 expression according to immunoreactivity score (IRS) into weak to no expression (0–5) *versus *moderate to strong expression (6–12). The complete statistical data are provided in Table 5. In Fisher's two-sided exact test, strong ITIH2 expression was highly significantly associated with presence of the estrogen receptor (p = 0.001).

**Table 5:** Clinicopathological and immunohistochemical parameters in relation to cytoplasmic ITIH2 immunoreactivity

**Variable**	**Categorization**	**ITIH2 immunoreactivity**				**Tumor-related death**			**Tumor recurrence**		
		
		**n analyzable**	**weak**	**strong**	**p^3^**	**n**	**events**	**p^4^**	**n**	**events**	**p^4^**
***Clinicopathological factor:***											
Tumor stage^1^											
	pT1	46	19	27	0.875	46	7	**<0.001**	45	10	**<0.001**
	pT2	92	43	49		92	32		89	41	
	pT3	13	5	8		13	6		12	6	
	pT4	30	12	18		30	19		27	19	
Lymph node status^1^											
	pN0	71	30	41	0.757	71	12	**<0.001**	69	14	**<0.001**
	pN1–3	104	47	57		104	47		100	57	
Histological grade											
	low grade (G1 – G2)	100	40	60	1.000	100	26	**<0.001**	95	33	**<0.001**
	high grade (G3)	80	38	42		80	38		78	42	
Histological type											
	ductal	145	61	84	0.915	145	51	0.772	142	65	0.371
	lobular	13	5	8		13	6		11	4	
	other	19	9	10		19	6		17	5	
											
***Immunohistochemistry (IHC):***											
Estrogen receptor status											
	negative (IRS^2 ^0–2)	49	30	19	**0.001**	49	21	0.080	49	25	0.096
	positive (IRS 3–12)	97	31	66		97	29		93	33	
Progesterone receptor status											
	negative (IRS^2 ^0–2)	107	44	63	0.862	107	48	**0.001**	101	50	**0.008**
	positive (IRS 3–12)	49	21	28		49	9		49	13	
HER2 expression status											
	negative	120	53	67	0.190	120	33	**<0.001**	113	43	**0.007**
	positive	38	12	26		38	22		38	22	

P-values according to Fisher's exact test (two-sided) were calculated for significance of correlation between clinicopathological and immunohistochemical parameters in relation to cytoplasmatic ITIH2 immunoreactivity. Furthermore, univariate analysis of factors regarding tumor-specific survival and recurrence-free survival was performed with significance calculated by log rank test (two-sided).

^1^According to UICC: TNM Classification of Malignant Tumours (2002) [65]. ^2^IRS = Immunoreactivity score according to Remmele and Stegner (1987) [47]. ^3^Fisher's exact test (two-sided). ^4^Log-rank test (two-sided). Bold face representing significant data (p < 0.05).

**Figure 5:**
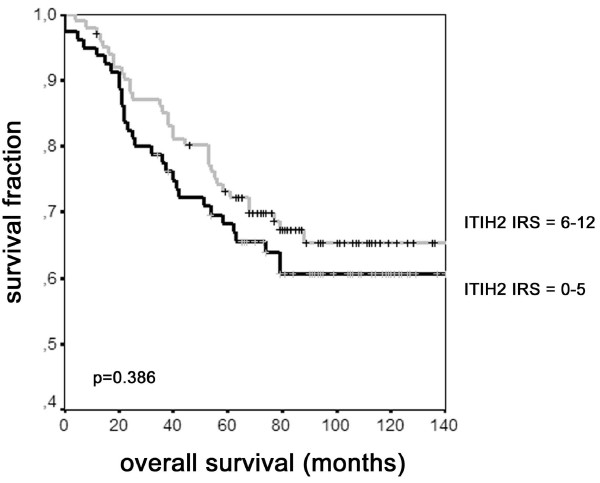
**Kaplan-Meier analysis of patients' overall survival (OS) with respect to ITIH2 expression status**. Cumulative survival is presented on the y-axis, tick marks represent censored patients.

Furthermore, overall survival (OS) and recurrence-free survival (RFS) were compared between tumors with weak *versus *tumors with strong immunoreactivity using univariate log-rank statistics in Kaplan-Meier analysis (Figure 5). Loss of ITIH2 expression was not significantly associated with shorter overall survival (p = 0.386) or recurrence-free survival (p = 0.948). Moreover, in a stratified Kaplan-Meier analysis, there was no significant association between loss of ITIH2 expression and shorter overall survival for patients with *versus *patients without lymph node invasion (p = 0.492 and p = 0.547, respectively), neither for ER-positive *versus *ER-negative patients (p = 0.358 and p = 0.359, respectively). Similarly, loss of ITIH2 expression was not significantly associated with shorter recurrence-free survival for patients with *versus *patients without lymph node invasion (p = 0.478 and p = 0.243, respectively), neither for ER-positive *versus *ER-negative patients (p = 0.312 and p = 0.931, respectively).

## Discussion

The family of inter-alpha-trypsin inhibitors (also named inter-alpha (globulin) inhibitors) is a long-known family of serine protease inhibitors, composed of a light chain with anti-proteolytic activity (bikunin) and different homologous heavy chains (ITIHs), which contribute to the stability of the extracellular matrix. There have been many studies on biological effects of the ITI molecules, proposing an involvement in various acute-phase processes, such as inflammation or cancer. However, there has been no approach to systematically identify expression patterns of ITIs in human tumors in order to reveal potential candidate tumor suppressor genes and/or oncogenes. In our present study, we address the issue of differential gene expression of the ITI genes, using Cancer Profiling Arrays containing spotted cDNAs from 241 tumor and 241 matched normal tissue samples representing 13 different human tumor entities. Therefore, in this study we present for the first time systematic expression patterns of *ITIH *genes in a broad panel of human normal and tumor tissue samples.

Our findings clearly show a strongly deregulated expression pattern in multiple human solid tumors for all *ITIH *genes except for *ITIH1*. In fact, deregulation exclusively presented as downregulation with different patterns for the *ITIH *genes in human tumor entities. Therefore, *ITIH *genes may represent putative tumor suppressor genes that should be studied in greater detail in further studies. Also, we clearly demonstrated that *ITIH *gene expression is not limited to liver as the main site of posttranslational modifications, although expression in liver by far exceeds mRNA quantities in any other tissue (except for *ITIH5*, which is most strongly expressed in placenta). These findings match the rare quantitative expression data of previous studies, using RT-PCR to determine expression levels of *ITIH *mRNA[49].

For a further analysis of ITIH expression on the protein level, we selected ITIH2 expression in normal and malignant breast tissue, since out of all ITIH family members, ITIH2 showed the most frequent downregulation (70%) in this tumor entity. Investigating *ITIH2 *expression in human breast cancer we were able to confirm the data obtained by CPA analysis (70% downregulation) by semiquantitative real-time RT-PCR (64% downregulation) in an additional set of breast tumors. Thus, CPA analysis proved to be a valid method for detecting differential gene expression in a systematic screening approach, as we were previously able to demonstrate[39,50]. Next, we analyzed for the first time ITIH2 protein expression in normal breast tissue and breast cancer. ITIH2 expression was highly significantly correlated with expression of the estrogen receptor (p = 0.001). Though estrogen is known to inhibit invasion and motility in breast cancer [51], the precise mechanism of this inhibitory effect is unknown. Since estrogen is known to influence extracellular matrix (ECM) integrity in responsive organs like uterus or vagina[52], estrogen could have a profound effect on ITIH molecules in normal and pathological breast tissues as well. *In silico *analysis of the *ITIH2 *gene promoter (-3500 to +200) revealed the presence of at least two estrogen receptor binding motifs (as predicted by Genomatix[53]), although the most common Estrogen Response Element (ERE) consensus sequence GGTCAnnnTGACC[54] was not found in this region. Nevertheless, Stender et al[55] showed that ER responsive MCF7 breast cancer cells respond to stimulation with ectopic estrogen by upregulation of *ITIH2 *expression in a time- and dose-dependent manner. This functional relationship is a subject of future studies to confirm our *in vivo *findings in an *in vitro *cell culture model. Also, it will be important to generate antibodies for the remaining ITIH family members in order to investigate their protein expression patterns as well as possible hormonal regulation in different human tumor entities.

In spite of a remarkably strong downregulation of the ITIH molecules in a variety of human solid tumors, we were not able to demonstrate a statistically significant association between loss of ITIH2 expression in breast cancer and reduced overall survival (p = 0.386) or shorter recurrence-free survival (p = 0.948) in Kaplan-Meier analysis. Likewise, there was no statistically significant correlation between loss of ITIH2 expression and reduced overall survival or recurrence-free survival, respectively, in a subgroup analysis for node status and estrogen receptor status. However, looking at expression patterns of the different heavy chain genes, there may be redundancy in their tumor suppressive functions, as e.g. ITIH2 and ITIH5 are both abundantly expressed in normal breast tissue. Recent studies in our workgroup revealed ITIH5 to be a novel prognostic marker in invasive node-negative breast cancer [37], proposing possible redundant functions of the structurally and genetically (*ITIH2 *and *ITIH5 *are both located on chromosome 10p15) related ITIH molecules. Altogether, the close association between ITIH2 and ITIH5, and their strong correlation with the estrogen receptor status, suggest that these molecules interact in their tumor-suppressive and metastasis-repressive properties.

Based on the knowledge of biological functions of ITIs, our data raise the hypothesis that these genes may exhibit tumor-suppressive properties on various levels of carcinogenesis. For many years, extracellular proteolysis has been known to be involved in development and spreading of cancer. However, the molecules contributing to the total proteolytic activity form a highly heterogenic group, to this date preventing efforts to present a wholistic concept. Even looking at subsets of proteases, such as the tissue kallikrein gene family, reveals the complex interaction of related enzymes[56]. Still, there is no question on the importance to understand and evaluate influences on extracellular proteolysis [57]. The ITI light chain, bikunin, has been identified previously to have anti-metastatic properties [58]). In particular, bikunin represses cell-bound plasmin activity [32,33] and is thought to inhibit CD44 dimerization and suppress the MAP kinase signalling cascade[58], thus preventing ECM degradation, tumor cell invasion, and angiogenesis.

Inhibition of tumor growth and spreading mediated by *ITIH *genes most likely relates to their stabilizing effects on the extracellular matrix, as well as their covalent linkage of hyaluronic acid (HA). In tissue remodelling, which is crucial to tumor growth and metastasis, hyaluronic acid turnover may play a key role. West and Kumar[59] reviewed the influence of hyaluronan on endothelial cells and neovascularization and concluded that HA degradation products may induce angiogenesis. Since HA linking and ECM stability is strongly dependent on ITI heavy chains, deregulation of *ITIH *family members should influence the vascularization process during tumor development. In particular, tumor suppressive effects have been attributed to the *ITIH *genes before: The short arm of chromosome 3 (3p), which ITIH1, ITIH3, and ITIH4 map to (see Table 1), is known to be a site of frequent genetic alterations in the evolvement of various human cancers (e.g. renal cell carcinoma[60], lung carcinoma[61], and others). In fact, in a survey on head and neck cancers, it has been proposed that the mapping site of ITIH1, 3 and 4 is a region which harbours several tumor-suppressor genes[62]. Furthermore, ITIH3 has been shown to be a downstream target of Sonic hedgehog (Shh)[63], which itself is know to be involved in pathogenesis of some human cancers, e.g. skin and brain cancers[64]. Investigating the interference of ITIH molecules and Shh may present a promising approach to elucidate functional interactions of ITI heavy chains. Finally, ITIH1 and ITIH3 have been shown to increase cell attachment and to reduce the number of lung cancer metastases in mice [36].

Altogether, our systematic ITIH expression analysis demonstrates that this gene family may harbour some promising new candidate tumor suppressor genes. Further studies will be needed to identify specific tumor entities and clinical settings, in which *ITIH *genes may serve as novel prognostic markers and possible therapeutic targets.

## Conclusion

In summary, our systematic analysis of differential gene expression in the family of inter-alpha-trypsin inhibitors leads to the following conclusions: *ITIH2*, *ITIH3*, *ITIH4*, and *ITIH5 *are strongly downregulated in a variety of human solid tumors (see Fig. 2, Table 4); therefore, the *ITIH *genes are potential candidates as tumor suppressor or metastasis repressor genes. Thus, ITIH molecules may serve as potential diagnostic markers or therapeutic targets in the malignant setting. Further studies will be needed to elucidate molecular pathways and biochemical interactions of the *ITIH *family, as well as their involvement in tumorigenesis and spreading of cancer.

## Competing interests

The author(s) declare that they have no competing interests.

## Authors' contributions

AH (Alexander Hamm) carried out the gene expression analyses, immunohistochemical studies, and statistical analysis, participated in the design of the study, and drafted the manuscript. JV processed clinical samples for PCR analysis, participated in the gene expression analysis and the design of the study, and helped to draft the manuscript. NB participated in immunohistochemical analysis, and critically revised the manuscript. PJW constructed the tissue microarray, provided clinicopathological data, and critically revised the manuscript. AH (Arndt Hartmann) participated in construction of the tissue microarray and collection of clinical data, and critically revised the manuscript. UH provided clinical samples for PCR analysis including clinical data, and critically revised the manuscript. GK participated in construction of the tissue microarray and collection of clinical data, and critically revised the manuscript. TWO generated the antibody for immunohistochemical analysis, and critically revised the manuscript. RDM participated in generating the antibody for immunohistochemical analysis, and critically revised the manuscript. RK participated in the design and coordination of the study, and critically revised the manuscript. ED planned and coordinated the study and critically revised the manuscript.

All authors read and approved the final manuscript.

## Pre-publication history

The pre-publication history for this paper can be accessed here:


